# A qualitative study to explore fathers’ attitudes towards breastfeeding in South West England

**DOI:** 10.1017/S1463423618000877

**Published:** 2019-03-20

**Authors:** Rowena Merritt, Michelle Vogel, Patrick Ladbury, Sally Johnson

**Affiliations:** 1 University of Oxford. Current: Centre for Health Services Studies, University of Kent, Canterbury, England; 2 Johns Hopkins University. Current: National Social Marketing Centre, London, England; 3 National Social Marketing Centre, London, England; 4 Wiltshire Council Public Health, Wiltshire, England

**Keywords:** breastfeeding, breastfeeding support, infant feeding, qualitative research

## Abstract

**Aim:**

To explore the beliefs, attitudes, and behaviours of fathers towards breastfeeding and how they impact either positively or negatively on their partners’ decisions to initiate or continue breastfeeding.

**Background:**

Despite policy initiatives at a national and international level and the increased number of baby-friendly hospitals within the UK, breastfeeding rates are slow to rise. Support from both parents has been proven to increase uptake and continuation rates, but there is little research into the emotional experience of fathers when it comes to breastfeeding.

**Methods:**

We conducted qualitative interviews with 18 fathers in Wiltshire, England. Principles of grounded theory were used throughout this study to guide the sampling, data collection, and data analysis.

**Findings:**

Fathers knew the health benefits of breastfeeding and wanted their child to breastfeed but were unsure of their place in the feeding process because they felt it was not their body. While they were aware of the benefits of breast milk for infants, fathers felt less informed of the practicalities of breastfeeding and the potential challenges they and their partner might have to overcome to breastfeed successfully for the recommended six-month period. Based on these findings, three segments were identified: the problem bonders, the dual bonders, and the pragmatists. All segments were concerned with the well-being of their partner and child and wanted their child to be breastfed. Health professionals can use the results of this study to create prenatal educational resources that take more of a preventive and problem-solving approach as opposed to promoting breastfeeding in efforts to comply with National Health Service guidelines, without offering solutions to common breastfeeding problems.

## Introduction

In the UK, breastfeeding is largely culturally accepted and promoted. Images of mothers breastfeeding their babies can be found in healthcare settings, parenting magazines, and other media emphasising the message that breastfeeding is healthier for babies (Lee and Furedi, 2005). Health professionals regularly communicate the benefits of breastfeeding to women during appointments pre-, during, and post-pregnancy. Maternity services in National Health Service (NHS) hospitals can earn the accreditation as ‘Baby Friendly’ by adopting UNICEF’s Ten Steps to Successful Breastfeeding (UNICEF, n.d.).

However, despite the policy initiatives at a national and international level, and the increased number of baby-friendly hospitals within the UK, breastfeeding rates are slow to rise. Socio-economic inequalities in breastfeeding remain, and across all the socio-economic groups the number of women who feed their babies formula milk remains large when set against the policy goal of six months exclusive breastfeeding (World Health Organization (WHO, n.d.; National Health Service (NHS), 2017).

Wiltshire is a rural county in South West England, with a strong military presence. In fact, the Army is Wiltshire’s biggest single employer. Military personnel constitute around 3.5% of the total population and the total number of military personnel and their dependants is estimated to be around 30,000 (6.4%). This is set to rise in the coming years as troops rebase in the UK as part of ‘Army 2020’ (Wiltshire Intelligence Network, 2012).

In Wiltshire, a higher proportion of women initiated breastfeeding (80.1%) compared with the Southwest overall (79.0%) and England overall (74.3%) in 2014/15. However, by six to eight weeks, only 49.4% of women were breastfeeding (Hodsdon, 2015).

Whilst there is a large body of evidence detailing the many different reasons why mothers do not breastfeed or discontinue breastfeeding before the recommended 6-month mark, there is little evidence detailing the emotional barriers and attitudes towards breastfeeding from the fathers’ perspective.

Most literature focused on fathers discussing antenatal education and concluded that the more fathers know about breastfeeding, the more likely they are to support it (Ingram and Johnson, 2004; Wolfberg *et al*., 2004; Pisacane *et al*., 2005; Tohotoa *et al*., 2009). Whilst the prenatal education does appear to work, there remain deeper, more emotionally-fed barriers to father’s continued support of breastfeeding. These include:

- the father’s desire to be a part of the feeding process (Earle, 2000; Rempel and Rempel, 2011);
- the desexualisation of the mother as a result of breastfeeding (Pontes *et al*., 2009; Henderson *et al*., 2011); and
- embarrassment of breastfeeding in public (Freed *et al*., 1992; Henderson *et al*., 2011).

Prenatal educational interventions seem to be key to initiation, but when it comes to continuation, the evidence is unclear. However, there is a growing body of research highlighting the influence of partners on breastfeeding. It is increasingly evident that the emotional, practical, and physical support of fathers is important in the promotion of successful breastfeeding and positively influencing the experience.

Wiltshire Council’s Public Health team commissioned the National Social Marketing Centre (NSMC), a not-for-profit organisation, to develop a social marketing intervention to positively influence the behaviour of fathers in relation to supporting their partners in breastfeeding. As part of the process, a study was conducted to examine fathers’ perceptions of breastfeeding and attitudes towards it.

## Methods

The aim of the research was to identify:

1. the beliefs, attitudes, and behaviours of husbands/partners in Wiltshire towards breastfeeding;
2. how current beliefs, attitudes, and behaviours of husbands/partners impact either positively or negatively on their partner’s decisions to initiate breastfeeding or the length of time they continue to breastfeed; and
3. ways in which husbands/partners prefer to receive key messages and/or information related to breastfeeding and how they prefer those messages to be framed.

As such, a series of qualitative interviews were conducted with fathers in Wiltshire.

### Recruitment and sampling

The participants for this study were selected using purposive sampling, meaning they were selected because they possessed knowledge that was directly related to the research questions (Lincoln and Guba, 1985; Patton, 2002). Once initial interest had been expressed by a potential participant, a short screening questionnaire was conducted. This was done to try and gain a greater mix of participants. Data gathered included (i) area they live in, (ii) age, (iii) number of children, (iv) employment status, (v) if more than one child, method of feeding done for previous newborns, and (vi) age of the youngest child (only those with a child under 12 months were eligible for the study).

This study aimed to reach *data saturation*, the point at which no new themes are identified and the emergent theory appears complete. It was of note, and unusual given the nature of the project, that many of the final themes were evident in the early stages of the interviewing process. Thus, although data saturation was reached early and after the first wave of interviews, further interviews were conducted to ensure the initial summation was accurate. Great efforts were also made by the research team to try and ensure a broad range of fathers, from different socio-economic backgrounds and of different ages, were interviewed. Fathers were told that the study was on infant feeding as opposed to breastfeeding in a bid to attract a wider range of participants.

### Participants were recruited through

- two local maternity wards,
- community midwives,
- posters in the local military bases and radio adverts on the military radio,
- local national childbirth trust groups, and
- snowball recruitment.

### Data collection

In-depth interviews were conducted with fathers. As the objective was to put participants at ease during the interview, fathers were offered the opportunity to be interviewed in their own home, over the telephone, or with their partner present (in a paired interview). Most of the fathers were interviewed individually, however five of the fathers preferred to be interviewed with the partner present, as they felt that breastfeeding was predominately the ‘*woman’s choice*’, therefore she should be included in any discussions around the topic area. To make the fathers feel at ease, paired interviews were then conducted, however it was stressed at the start of the interview that the purpose was to understand the *father’s* perspective and if the mother started to lead the conversation, the interviewer carefully facilitated to ensure that the father was the focus of any discussions.

All participants were interviewed once between May 2014 and August 2014. The median length of an interview was 47 min. Interviews were recorded. Participants were informed of the intention to record in the study information sheet and the recorder was placed in a position clearly visible to each participant. Verbal informed consent was sought and obtained from all participants.

#### Interview guide

Semi-structured interviews were conducted and at the start of each interview loosely structured, open-ended questions were asked. The wording for the questionnaire was not standardised as the interviewer tried to use the participant’s own vocabulary to guide the discussion. Questions asked covered multiple domains consisting of:

- feeding plans and knowledge around breastfeeding,
- attitudes towards breastfeeding and perception of benefits,
- perceived role in feeding,
- experience of breastfeeding,
- confronting challenges in breastfeeding, and
- informational and communication needs.

### Data analysis

All the interviews were transcribed verbatim. The transcripts used accepted procedures for indicating exclamations, pauses, and emotion (Field and Morse, 1985; Seal *et al*., 1998). Transcriptions were imported into NVivo (QSR International Pty Ltd, 2012).

Analysis of the interviews used the constant comparative method. Two researchers conducted the analysis and coded the data separately at first and then together where they compared their coding and finalised the analysis work.

Only data from the fathers was included in the analysis. The transcripts from the paired interviews were also carefully reviewed to ensure that having the mother present did not alter the answers, which it appeared not to do.

In discussing the results, using quantitative descriptions to describe qualitative data seemed inappropriate, therefore, frequency of a response is indicated by such terms as, ‘all’, ‘most’, ‘many,’ ‘some’, ‘a few’, or ‘one’.

## Results

The study sample consisted of 18 men. All participants classed themselves as ‘White British’, except for four participants who identified as ‘White Other’. Most of the participants had a university degree (*n*=13). Only one classed himself as being unemployed. Four of the men interviewed worked for the armed forces.

The mean age was 33.6 years (range 21–42 years). The mean age of the youngest child was four months (range 5 days–11 months). In relation to feeding practices:

- Nine of the babies were mixed fed, most from birth (or within the four weeks of being born).
- Two were breastfed exclusively until 6 months.
- Two were breastfed exclusively until 5 months.
- Eight were still being breastfed at the time of the interview (all of these babies were under six months old).
- Two were formula fed from birth.

Seven of the fathers interviewed had other children.

### Knowledge and attitudes towards breastfeeding

Most of the fathers knew ‘*nothing*’ about breastfeeding before their child was born. Many of the fathers were surprised to learn how difficult and painful breastfeeding could be for some mothers.

*I thought it would just come naturally. I didn’t realise it’s not always the case. It seems the most natural thing in the world, but still so many mothers struggle at first.*

All fathers believed breast milk was best for the baby, and this view was held by most, even before attending any prenatal appointments or classes because it was seen as ‘*natural*’.

*I suppose regardless of financial bits there’s just the natural process of it and clearly all those natural ingredients – proteins and god only knows what’s in that natural product – rather than something that’s bought off the shelf.*

*Really the immune system and the nutrition you get from breast milk and also the thought of what’s in the formula as a source of soy and fish and disgusting artificial things, I just don’t like the thought of it at all.*

Some of the fathers also talked about the emotional bonding gained by breastfeeding.

*I think in a really positive way because there’s something wonderful about seeing your wife have that emotional attachment to your child….*

This bond between mother and child was regarded by most of the fathers as a ‘*beautiful thing*’. However, some fathers did admit that they missed having that same bond with the child.

Nearly all of the fathers talked positively about feeding their child a bottle. Sometimes this was a bottle of expressed milk, other times formula milk. The fathers really enjoyed having this intimate time with their child. As one father said:

*I loved giving [son’s name] a bottle. I felt like I was giving him some pleasure. When my wife was breastfeeding I just did all the things our son didn’t like – changing his nappy, bathing him, and so on. So when I was feeding, it felt like I could do something nice for him, something he actually wanted.*

Giving the baby a bottle was regarded by most of the fathers as the way to be ‘*intimate*’ and have ‘*bonding time*’ with the baby. In fact for a few fathers, not being able to bottle feed their child made bonding more difficult.

*I know I shouldn’t have felt like this, but until my wife started mixed feeding I…I felt, and I know again I shouldn’t have felt like that, but I felt removed from the situation. I didn’t bond with our son straight away. But when I started giving him a bottle … yes I think that helped.*

### The mother’s wishes

Most of the fathers postulated that it was the mother’s decision whether to breastfeed or not. Only one of the fathers was adamant that their partner breastfeed. With many of the fathers, it was discussed only vaguely and most of the fathers said it was something they thought their partner would ‘*just want to do anyway*’.

*I don’t think we actually discussed it, it was just kind of a given I suppose really….*

Nearly all of the fathers stressed that they would have supported their partners either way. They also felt that they could, and should *not*, influence the decision as it was their partners ‘*breasts and body*’ and their ‘*decision to make*’. This finding differs from how men are often portrayed in the media and otherwise as wanting to control their partners or partners’ bodies.

*As a man I’ve got to step back on a lot of this stuff and not make a decision and just go with what [partner’s name] believes is best. Mum knows best.*

### Conflicting advice

When asked about support received from health professionals in relation to breastfeeding, many of the fathers had very strong views on the advice they had been given. Frequently the women had experienced difficulty with breastfeeding. During this time, various health professionals (usually midwives) had been consulted and advice given. This advice was often felt to be contradictory. For example, one midwife might suggest one feeding position or technique, then the next day another midwife would suggest something totally different and tell the parents that it was a bad position they had been trying before. Again, this led to further frustration for the father and the feeling that the health professionals (in particular midwives and health visitors) were being too ‘*pushy*’ and not thinking ‘*about the mother*’.

*And it did, you know, the midwives kept saying, ‘Oh try this, try that,’ and I was actually getting quite angry because I was thinking she’s tried everything and, you know, the stuff that you’re coming out with she’s already tried. And this is another thing; midwives say different things.*

A few of the fathers felt that the health professionals had not given them the correct advice as they ‘*had to keep within NHS guidelines*’. This was mentioned predominantly in relation to the nipple shields.

*… one of the NHS midwives who went completely by the rule book said, ‘I’m not allowed to recommend this to you because it’s not in our guidelines but I think if you pop to the pharmacy and get some nipple shields he’ll probably breastfeed.’ So I went down to the pharmacy, got some nipple shields and low and behold 3 seconds later he was breastfeeding … this was after four days of struggling!*

### Information specifically for fathers

Fathers were asked whether they had received any specific materials or information designed explicitly for fathers. None of the fathers had received any information about breastfeeding or fatherhood in general. Fathers were polarised in their views in relation to father-specific information around breastfeeding. Whilst some felt it would be useful, in particular information on how to support their partner and different breastfeeding positions, others believed that information around breastfeeding should always be focused towards the mother, as she would be the one doing it.

*How to cope when things go wrong … I would have read that.*

### Difficulty breastfeeding

For many of the fathers interviewed, their partner had often experienced difficulties at first with breastfeeding. For most, when their partner experienced the problems, they were initially shocked, as they had never thought that ‘*such a natural process*’ would cause any difficulties. Having to watch their partner in pain or distress was very upsetting for the fathers, and they reported a feeling of helplessness, which in turn lead to frustration and often anger.

*I think it’s best sometimes to paint the worst possible picture and I think in a lot of things, especially breastfeeding, they [NCT classes] just paint the best possible picture. You know; it’ll be great and it’s really good for them and there’s lots of health benefits but actually I kind of wish she was prepared for it because I hated sitting there thinking well there’s nothing I can do.*

A few of the fathers felt that the health professionals had ‘*painted a rosy picture*’ of breastfeeding and that for some women, this was not the reality. Therefore, when the problems arose they had no idea how to handle the situation. In addition, when some of the women started to mix feed or express (often on reported advice from the health professional), the fathers reported that they had no idea how to sterilise bottles or prepare formula milk as this had rarely been covered in the prenatal classes.

*Of course as a new mum who’s having her first child who’s exhausted, she’s tired, she’s emotional anyway and then people are telling you, ‘You’ve got to get your baby feeding,’ and you’re like, ‘Well he won’t take it.’ And no one prepares anyone for that.*

### Support given

Most of the fathers were concerned with their partner’s well-being (often mental well-being), and a few felt that health professionals should consult with them more and discuss the difficulties with them about well-being and breastfeeding, as they were the ones who knew their partners the best. However, when it came to breastfeeding, they were rarely consulted or even spoken to about the topic area.

*I don’t think I was ever included in any of the breastfeeding conversations … often when I was in the room – I was never asked how it was affecting my wife.*

All of the fathers talked about ways in which they had supported their partners whilst they were breastfeeding. They were anxious to help their partners as they were very conscious of the strain breastfeeding placed on their partners. Support was given in various ways, including:

- encouraging the mothers not to give up and providing moral support,
- helping with positioning,
- burping and nappy changing, and
- doing household chores and looking after the other children.

Many of the fathers, whose partners had experienced difficulties, discussed how they had supported their partner. This was often by providing moral support, especially in the early hours when their partners had often felt most alone.

*I think you’ve just got to be there … Just being there, even if all I do is get the feeding cushion and turn it around or something or I’m just there awake ….*

### Encouraging their partner to initiate or keep breastfeeding

As discussed previously, most of the fathers believed that it was the women’s choice whether they breastfeed or not, and that, as the father they had limited say and their role was just to support their partner. Therefore, most of the fathers said they had no idea what they could do or say, or even if they had the right to say anything.

*I … umm … I have no idea as, well it’s up to her really. She would feel guilty anyway as she knows breast is best and maybe after a while, she would just want her body back to herself. It’s really intense breastfeeding.*

*It’s not really my place to put more pressure on her.*

A couple of the men felt that they could suggest mixed feeding; the mother would be able to rest more as they could help with the feeding and the baby would still get the health benefits of breastfeeding.

*I think the NHS promote breastfeeding so vigorously that women do not realise you can do both [bottle and breast] maybe. Among our friends, they seem to think it’s all or nothing.*

## Discussion

Even though past research has shown the importance of support from the father in initiating and continuing breastfeeding, most of the fathers in this study did not believe that they could, or should, influence their partner’s decision on whether to breastfeed or not. Even if they would have preferred for their baby to be breastfed, they did not feel it was their right to insist or, in some cases, even comment around the subject matter. It is possible the participants expressed these opinions because they did not want to seem controlling to the researcher in light of public discourse that men should not control women’s bodies. However, it is not clear whether this bias exists in the data or not. Sherriff and Hall (2011) acknowledged that public discourse on men could also influence health professionals in their efforts to include men in the conversation and education about breastfeeding. That is, health professionals may avoid engaging men in the discussions they have with women about breastfeeding to avoid seeming like they are facilitating men controlling women’s bodies.

Some participants seemed surprised that we even wanted to interview them on this subject. For example, when the fathers were asked whether they had any questions before the interview started, a few asked whether their views and opinions really mattered.

Interviewer: *Have you any questions before we begin?*

Participant: *‘Do we matter’ is the first question to you?*

Interviewer: *Well you matter very much to me! I would like to know your views.*

This quote and similar questions posed by other fathers was indicative of the view that many of the fathers shared – that it is the woman’s prerogative to breastfeed or not.

That said, all of the fathers in this study preferred breast milk to formula as it was regarded as a ‘*natural*’ substance and therefore was seen as healthier for the baby. Fathers did appear to have an opinion on what they wanted to feed their child and were aware of the many health benefits a child could receive from being fed breast milk.

The literature consistently shows a strong connection between the father’s emotional support and the mother’s initiation and/or continuation of breastfeeding (Arora *et al*., 2000; Rempel and Rempel, 2011). Thus, there is still room for health practitioners to further engage fathers during the pregnancy about breastfeeding. The fathers often attended the prenatal classes and/or the hospital appointments and whilst in attendance, health professionals could openly ask the fathers whether they want their child to be breastfed or not.

Also, when attending house visits in the early days after discharge from hospitals, fathers could be consulted and their options sought. This is particularly valuable if the mother eventually struggles with breastfeeding. Arora *et al.* showed that it is the mother’s perceived support from the father as opposed to actual support or the father’s perceived support that played a role for mothers in continuing to breastfeed (2000). By eliciting the support early on, there is an opportunity to build the mother’s perceived support from the father in her breastfeeding endeavours.

The findings of this study support previous studies in their assertion that there remains a strong need for quality educational and informational resources targeted at fathers during pregnancy (Arora *et al*., 2000; Wolfberg *et al*., 2004; Pisacane *et al*., 2005; Tohotoa *et al*., 2009; Sherriff and Hall, 2011; Brown *et al*., 2014). A randomised controlled trial in Australia showed that even just a 2-h hospital-based antenatal session and postnatal support for fathers significantly improved breastfeeding rates at six weeks follow-up when compared to a control group (Maycock *et al*., 2013). Similarly, a qualitative study on the effect of trained maternity care assistants giving an antenatal intervention with fathers and mothers together was well-received. Fathers felt more prepared to support their partner in breastfeeding after the intervention (Ingram and Johnson, 2009).

This research proposes that prenatal classes should include the following practical information:

- Hands-on experiences, such as practice at changing diapers and putting a baby into the right positions for feeding. Fathers felt there was a lack of practical information around the preparation of bottles and the sterilisation of breast pumps.
- An honest view of breastfeeding. Many of the fathers criticised the prenatal classes as they believed they painted an unrealistic picture of breastfeeding to further their agenda of increasing breastfeeding rates. They would prefer if classes explained that problems could arise and provided practical solutions to try when they do.

Overall, this study showed that the support a father can give the mother when they are struggling with breastfeeding is immense. However, fathers are often unaware of the problems that can arise and then feel frustrated and unable to help. They also are not sure of their role in feeding decisions and the feeding process. These points echo a previous study with men whose partner had given birth within the past two years; researchers found fathers wanted to support their partner in breastfeeding but felt left out of any discussions with health professionals or antenatal education on the matter (Brown *et al*., 2014). Similarly in a study including in-depth interviews of fathers in the UK, researchers found fathers wanted to be more involved in breastfeeding but felt there was a lack of information around the benefits and practicalities of breastfeeding targeted at them (Sherriff and Hall, 2011).

Fathers were often angry with the health professionals who they felt did not look at their partners’ well-being and were too focused on following the NHS guidelines. Therefore, fathers should be included in all feeding discussions by health professionals before, during, and after pregnancy. They should be asked their opinion, informed of some of the issues that may occur, and of what practical, as well as emotional support they can give their partner during this time.

### The joy of feeding a baby and decision-making

Most of the fathers talked about the joy of feeding their child and the bond that came with this type of intimacy, as in many previous studies (Earle, 2000; Earle, 2002; Rempel and Rempel, 2011). Although the fathers loved to see their partners’ bond developed through breastfeeding, they also wanted to have that closeness with the child themselves.

As this emotional aspect of feeding plays such a large role, there should be more information targeted to fathers about other ways they can gain that emotional bond, for example, skin-to-skin.

Most couples did not actively sit down and discuss whether to breastfeed their child. It was usually a decision made by the mother and then talked about with the father after the decision had been made. Decisions about breastfeeding are known to be made before conception or in the early weeks of pregnancy (Earle, 2000).

### Development of a segmentation model

Based on the findings from the interviews, a segmentation model was developed. In social marketing, segmentation is based on psychographics and behavioural characteristics, as opposed to solely demographics or geography.

The development of a segmentation model was difficult to do for this project as most of the fathers had very similar values and attitudes towards breastfeeding, wanting the best outcomes for both their partner and child. The segmentation was developed based on the fathers’ feelings towards feeding their child in relation to how it made the fathers feel. These are not ‘static’ segments, and people can move from one segment to the other.

#### Segment 1: the problem bonders

Fathers in Segment 1 often experience bonding issues with their child. This is usually with the first child, however, it can be experienced for the first time with subsequent children as well. The fathers often feel overwhelmed and struggle to cope with the arrival of their child. However, whilst feeling overwhelmed, they also feel underwhelmed by the experience. Often these feelings develop once the child is out of hospital and they must cope with changes to their day-to-day routines. They feel ‘lost’ and stressed out by all the new activities they need to learn, such as changing nappies and burping their child. All these factors mean that they struggle to bond with their child, which they feel guilty about. They find that feeding their child a bottle, holding them close and nurturing them helps them to develop a greater bond. They feel guilty for feeling this way and still believe breast milk is the best for the child.

#### Segment 2: the dual bonders

Segment 2 also enjoys giving their child a bottle; they love the intimacy and the time it gives them with their baby. However, they also love to watch the bond developing between mother and child and the emotional attachment that comes from breastfeeding. They like to think of themselves as being very hands-on and help with as many of the chores and jobs around the house as possible. As with the other segments, they feel it is the mother’s choice whether to breastfeed or not and feel strongly that breastfeeding is the best start for their child. They are the segment most likely to attend National Childbirth Trust classes and to suggest different positions for the mother to try to help with the breastfeeding; they encourage their partner not to give up by giving moral support, often during the early hours.

#### Segment 3: the pragmatists

Whilst Segment 3 individuals also believe that breastfeeding is the best start in life and want their partner to breastfeed, if their partner was struggling, they were more likely to suggest giving the baby a bottle of formula milk. They appeared to be more indifferent about giving the baby a bottle and did not mind if they fed their baby or not. They took a very pragmatic approach to problems and felt that the baby just needed to be fed, and it did not really interest them how this was done. They were the least likely to attend prenatal classes with their partners as they took a very laid back approach to everything, concluding that they would be forced to learn everything quickly enough when the baby was born.

### Recommended interventions

Based on the research findings and the segments identified, a number of recommendations were made to Wiltshire Public Health team (Table 1). All of the interventions recommended appealed to *at least one* of the segments. The recommendations were divided into those that could be potentially delivered in the short-, medium-, or long-term.

**Table 1:** Recommendations

Time period	Recommendation
Short-term (0–3 months)	∙ Encourage midwifes to also involve fathers in the discussions at antenatal appointments ∙ Change the conversation to include mothers and fathers in relation to feeding decisions before the birth, e.g., what have you both discussed/decided in relation to feeding your baby? And changing the message ‘the way that is right for you’ ∙ Provide information on where they can access breastfeeding support information to both mothers and fathers when leaving hospital ∙ Encourage skin-to-skin with fathers post-birth
Medium-term (6–12 months)	∙ Develop an electronic ‘DadPad’ for Wiltshire to provide practical information and tips tailored to fathers ∙ Address invitation letters to attend appointments to both the mother and the father ∙ Offer practical sessions for fathers on how to support a partner who is breastfeeding in antenatal classes
Longer-term (12 months+)	∙ More flexible working to enable evening and weekend visits by healthcare professionals to ensure fathers with less flexible working patterns can also be involved. ∙ Better use of technology to enable a variety of communication methods, e.g., Skype, texting to help answer concerns/questions quickly

### Strengths, limitations, and recommendations for further research

The findings from this study can be used to inform current best practices for helping families start and continue to breastfeed. This study was limited in that most of the participants came from middle and high socio-economic backgrounds, despite efforts to recruit from lower strata. The fathers’ views thus do not reflect lower-income populations in the region.

There is a gap in the research in terms of non-educational interventions that help mothers start and continue breastfeeding that are aimed at the father. Knowledge does not necessarily mean behaviour change, so further research could test other types of interventions to reduce barriers to breastfeeding uptake and continuation.
